# The 3D Printing and Evaluation of Surgical Guides with an Incorporated Irrigation Channel for Dental Implant Placement

**DOI:** 10.3390/bioengineering10101168

**Published:** 2023-10-07

**Authors:** Robert-Angelo Tuce, Monica Neagu, Vasile Pupazan, Adrian Neagu, Stelian Arjoca

**Affiliations:** 1 Department of Functional Sciences, Victor Babes University of Medicine and Pharmacy Timisoara, 300041 Timisoara, Romania; tuce.robert@gmail.com (R.-A.T.); neagu.monica@umft.ro (M.N.); pupazan.vasile@umft.ro (V.P.); arjoca.stelian@umft.ro (S.A.); 2 Center for Modeling Biological Systems and Data Analysis, Victor Babes University of Medicine and Pharmacy Timisoara, 300041 Timisoara, Romania; 3 Department of Physics and Astronomy, University of Missouri, Columbia, MO 65211, USA

**Keywords:** osteotomy, surgical drilling, surgical templates, intraosseous temperature, cooling

## Abstract

Dental implant insertion requires the preparation of the implant bed via surgical drilling. During this stage, irrigation is essential to avoid thermal damage to the surrounding bone. Surgical guides enhance the accuracy of the implant site preparation, but they mask the drilling site, hampering coolant delivery. A variety of designs are aimed at improving the coolant access to the target site. Using standard dental implant simulation software, this paper presents an in-house design and 3D printing workflow for building surgical guides that incorporate a coolant channel directed toward the entry point of the burr. The proposed design was evaluated in terms of the bone temperature elevations caused by drilling performed at 1500 rpm, under an axial load of 2 kg, and irrigation with 40 mL/min of saline solution at 25 °C. Temperature measurements were performed on porcine femoral pieces, in the middle of the cortical bone layer, at 1 mm from the edge of the osteotomy. The mean temperature rise was 3.2 °C for a cylindrical sleeve guide, 2.7 °C for a C-shaped open-sleeve guide, and 2.1 °C for the guide with an incorporated coolant channel. According to a one-way ANOVA, the differences between these means were marginally insignificant (*p* = 0.056). The individual values of the peak temperature change remained below the bone damage threshold (10 °C) in all cases. Remarkably, the distribution of the recorded temperatures was the narrowest for the guide with internal irrigation, suggesting that, besides the most effective cooling, it provides the most precise control of the intraosseous temperature. Further studies could test different design variants, experimental models (including live animals), and might involve computer simulations of the bone temperature field.

## 1. Introduction

Before dental implant insertion, a cylindrical cavity needs to be prepared in the target site via surgical drilling [1]. The prepared cavity enables the insertion of the dental implant, at a moderate torque, without further damage to the surrounding bony structures. Excessive heat generation during this process can lead to bone damage, impairing the osseointegration of the implant [2].

The elevated temperatures reached during bone drilling have been of concern since the 1950s [3,4]. Vital microscopy revealed that 47 °C is the threshold temperature for bone tissue damage [5]. One-minute exposure to this temperature leads to minor bone resorption that is hard to differentiate from normal bone remodeling. Nevertheless, the microvasculature becomes dilated and remains so for about 5 days, and part of the fat cells present in the heated area die. When the exposure time increases to 5 min, about 20–30% of the bone tissue becomes resorbed and replaced by fat cells within one month. When bone is heated to 50 °C for one minute, most fat cells die and about 30% of the bone is lost and replaced by fat cells that invade the damaged area. At 53 °C applied for one minute, the blood flow stops in the heated area; the original blood vessels die within days and become gradually replaced by ingrowing capillaries [6]. At 56 °C, alkaline phosphatase is denatured. At 60 °C, the bone tissue becomes necrotic, unable to recover for months or even years [5]. Even brief exposure, of the order of seconds, to temperatures of 90 °C or higher, results in bone necrosis [7].

The temperature rise during implant site preparation depends on several factors related to the employed instruments, the adopted methodology, and patient characteristics. Among them, the most influential factors are (i) the drilling speed, (ii) the force applied by the surgeon, (iii) the cooling method, (iv) the drill’s wear, (v) the drill’s design, material, and diameter [8], (vi) the drilling procedure (guided or free-hand, single or multi-stage), and (vii) the bone mineral density in the target site [9].

Investigations of the intraosseous temperature elevation during guided implant placement have gathered momentum in recent years [10,11,12,13,14,15,16,17,18,19,20,21,22,23,24,25,26]. Bone cooling during surgical drilling is most commonly achieved by directing a stream of physiological saline solution onto the target site. To be effective, the irrigating fluid jet should reach the point of the penetration of the drill bit [27]. Therefore, surgical guides might interfere with effective cooling, since they mask the target site and divert the coolant jet. Indeed, the most common surgical guide (a splint with a cylindrical sleeve) practically blocks the access of the irrigating fluid, resulting in a significantly higher temperature rise than that observed in a conventional, free-hand osteotomy [15].

Despite the above concern, the use of surgical templates is becoming increasingly popular, because it assures more accurate results than the conventional, free-hand method [28,29]. Indeed, in guided implant insertion, the average distance between the planned and actual positions was found to be below 1 mm at both the implant entry point and the apex. In contrast, during free-hand implantation, the mean error at the apex was 2.5 mm along the medio-lateral axis and 2 mm along the antero-posterior axis; at the base, these errors were even larger: 3.5 mm and 2.4 mm, respectively. The average deviation of the implant axis from its planned direction was 4.2° when surgical guides were used and 9.8° otherwise [28]. A meta-analysis of 14 clinical studies on guided implant placement indicated a mean deviation of 1.25 mm at the entry point and 1.57 mm at the apex, and a mean angular deviation of 4.1° [29]. Nevertheless, significant differences were found between different techniques. Totally guided surgery (in which the surgical guide is used both for the implant site preparation and implant placement) was more accurate than partially guided surgery (in which the surgical template is only used for the osteotomy while the implant is inserted conventionally). In addition, the flapless procedure (drilling and insertion through the intact soft tissue) proved to be more accurate than the open-flap approach (in which the soft tissue is temporarily removed and the implant is inserted into the exposed alveolar bone) [29].

Several studies have indicated that the bone temperature remains in the safe zone during guided osteotomy performed with external cooling. In their pioneering study [15], Misir et al. demonstrated that surgical guides can elicit a statistically significant increase in the bone temperature elevation caused by implant site drilling. Osteotomies performed on bovine femoral cortical bone pieces resulted in a mean temperature rise of 39.7 °C when surgical guides were used, and only 30.7 °C when drilling was performed in their absence—as indicated by a thermocouple placed at 1 mm from the osteotomy edge, 6 mm beneath the bone surface [15]. Working on porcine ribs covered by a wax layer to simulate soft tissue, Migliorati et al. compared the drilling-associated temperature increments generated under four different conditions [19]: open-flap standard surgery, flapless standard surgery, open-flap guided surgery, and flapless guided surgery; the medians of the corresponding temperature increments were 1.25 °C, 1.42 °C, 4.40 °C, and 4.95 °C, respectively. The in vivo study conducted by dos Santos et al. on surgically exposed rabbit tibia revealed statistically significant differences between the maximum bone temperatures reached in the presence and the absence of osteotomy templates (31.8 °C and 28.5 °C, respectively) [20]. Boa et al. measured the peak temperature rises during flapless guided surgical drilling performed on bovine rib cortical bone specimens [18]. They found mean temperature rises of up to 5.2 °C when a jet of saline solution at room temperature irrigated the drill’s point of entry into the metal sleeve of the canal of the surgical guide. Moreover, out of 168 osteotomies, only 1 outlier resulted in a temperature rise higher than the critical 10 °C threshold [18]. Although the temperature increments in these studies remained below the bone damage threshold, they should be treated with caution because of differences in the material properties between human tissues and those used in the experimental models [19].

To further optimize the bone temperature regime during the preparation of dental implant sites, recent studies have evaluated the impact of the surgical template design on the temperature rise caused by guided osteotomy. Waltenberger et al. tested five template designs based on a carefully standardized bovine rib osteotomy protocol [16]. They found mean temperature elevations of, at most, 5.6 °C, and no statistically significant differences between different study groups. Choi et al. compared surgical guides with and without incorporated metal sleeves [11]. While metal sleeves ensure a better accuracy [30], it was hypothesized that they cause additional tissue heating due to friction with the drill, but their results did not confirm this conjecture [11]. Ashry et al. evaluated surgical templates with (i) classical, cylindrical sleeves, (ii) C-shaped open sleeves, and (iii) cylindrical sleeves modified to include lateral holes and a semi-cylindrical canal running along their periphery. Compared to the cylindrical sleeve, both the open-sleeve and the modified cylindrical sleeve ensured statistically significant reductions in the peak intraosseous temperatures reached during surgical drilling [21].

Most surgical template designs investigated to date have focused on facilitating the access of the irrigating fluid instead of guiding it toward the target site. The incorporation of an irrigation fluid duct into a surgical drill guide was first proposed by Liu et al. [31] and resulted in a 49% reduction in the peak temperature increment compared to conventional cooling. Their design, however, was limited to a surgical guide with regular geometry. Tuce et al. proposed a design procedure for creating a 3D printed surgical template with an incorporated coolant tube suitable for dental implant site preparation. The tube was included in the digital design as a simulated custom implant oriented along the desired irrigation direction [32]. Alevizakos et al. reported a case study using a surgical template with internal cooling ensured by a duct that transported the irrigation fluid to the entry point of the burr [23]. Orgev et al. took advantage of the design features present in most dental implant planning software (the ability to include fixation pins) to create a clinically applicable surgical template augmented with an auxiliary irrigation channel [22]. Nevertheless, less attention has been paid to measurements of the intraosseous temperature increments caused by osteotomy guided by templates with built-in irrigation pipes. To our knowledge, this problem has been addressed by three studies so far [24,25,26]. Stocchero et al. tested surgical guides with internal cooling on bovine rib specimens and did not find any benefits compared to classical guides [25]. Teich et al., on the other hand, demonstrated significantly more effective cooling when the irrigation fluid was guided toward the osteotomy site via a pair of internal irrigation fluid channels [26]. Parvizi et al. conceived an internally cooled surgical guide that incorporated both an entry and exit channel for the cooling agent and found a significant reduction in the intraosseous temperature [24] within the experimental framework employed previously by Stocchero et al. [25].

Therefore, the present study aimed to design a surgical guide that incorporates a coolant channel directed toward the entry point of the drill bit into the target tissues. The primary objective of the present study was to develop an in-house design and 3D printing workflow, using a standard dental implant planning software, to ensure that the new design can be fitted to any particular anatomy (e.g., to a given patient or experimental model system). Furthermore, we aimed to evaluate the proposed design concerning the bone temperature elevation caused by surgical drilling and compare it with the cylindrical sleeve and open-sleeve designs.

## 2. Materials and Methods

### 2.1. Design and Fabrication of Surgical Guides

For this study, we designed three types of surgical guides: (i) a classical guide, which includes a single cylindrical orifice that guides the drill, (ii) an open-sleeve guide, which includes a cylindrical orifice with a longitudinal slit that enables the lateral access of the drill into the guiding channel, and (iii) a guide that incorporates a coolant pipe, which can be coupled to the irrigation tubing and transports the coolant towards the drilling site. The latter will be called hereafter a surgical guide with auxiliary cooling.

The surgical guides were designed using the Blue Sky Plan software (Blue Sky Bio, Libertyville, IL, USA). To adapt the design for the subsequent thermodynamic testing, we scanned a piece of porcine femur with a Pax-i3d cone-beam computed tomography (CBCT) device (Vatech, Hwaseong, Republic of Korea).

**Figure 1:**
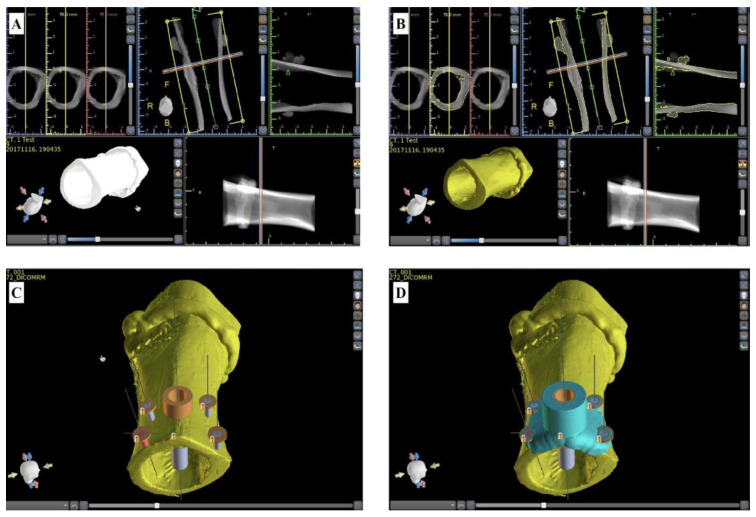
Snapshots of the Blue Sky Plan software window. (**A**) The CBCT-derived DICOM image of a porcine femur piece; (**B**) the 3D model of the femur; (**C**) the 5 implants simulated for delimiting the orifices of the osteotomy guide (central implant) and the holes needed for the immobilization of the surgical guide (peripheral implants); and (**D**) 3D model of the surgical guide (blue) fixed on the femur piece (yellow).

The Digital Imaging and Communications in Medicine (DICOM) file produced by the CBCT scanner was imported into Blue Sky Plan (Figure 1A).

The snapshots depicted in Figure 1 show the design steps that led to the 3D model of a surgical guide fitted on the underlying bone. Based on the CBCT image of the femoral piece (Figure 1A), its 3D model was created in Blue Sky Plan (Figure 1B). It was outfitted with a simulated dental implant (4.5 mm in diameter and 12 mm in length) oriented perpendicularly to the bone surface; its axis was placed at a distance of 5.25 mm from the bone margin (Figure 1C). Custom implants with a diameter of 1.5 mm and length of 5 mm were simulated in the construct’s corners (Figure 1C, blue cylinders) for delimiting the orifices needed for the wires that fixed the guide on the bone surface (Figure 1D; wires not shown).

**Figure 2:**
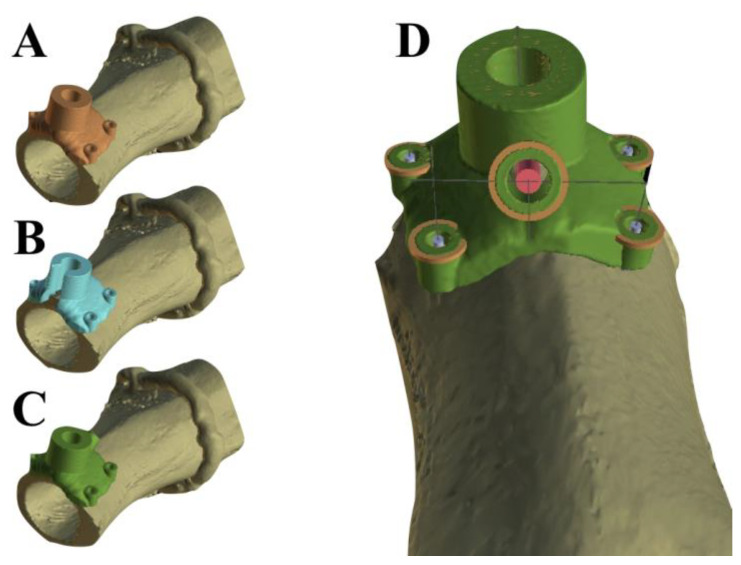
Design of the surgical guides used in this study. (**A**) The classical guide; (**B**) the open-sleeve guide; and (**C**,**D**) different perspectives of the guide with auxiliary cooling.

For the classical guide (Figure 2A), we followed the steps explained in Figure 1. To create the open-sleeve guide, we replicated the 3D model of the standard guide and utilized the “Cut” function in Blue Sky Plan to generate a longitudinal slit with a radial opening of 70 degrees (Figure 2B). Finally, the classical design was fitted with a coolant transport channel by simulating a custom implant (2 mm in diameter and 10 mm in length) oriented towards the osteotomy site (Figure 2D, pink cylinder) to delimit the coolant transport channel.

After exporting the digital models of the three guides as standard tessellation language (STL) files, we employed a Duplicator7 digital light processing (DLP) printer (Wanhao, Jinhua, China) to physically create them. The material of choice for the 3D printing was NextDent SG dental resin (NextDent, Utrecht, The Netherlands).

### 2.2. Experimental Setup for the Evaluation of the Cooling Efficacy

**Figure 3:**
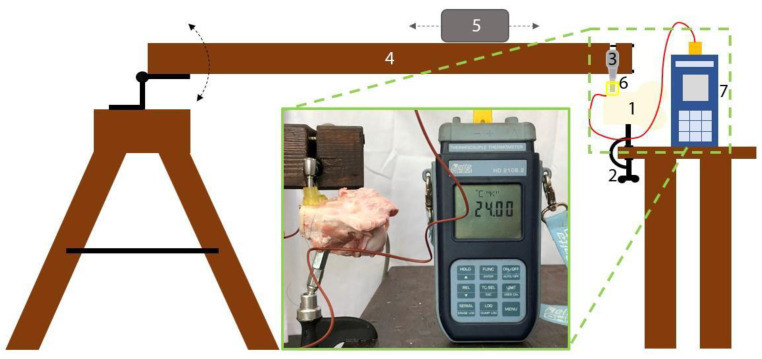
Schematic representation of the used experimental setup: (1) bone pieces, (2) bone stand, (3) handpiece, (4) rotating arm, (5) sliding weight, (6) surgical guides, and (7) digital thermometer. The inset shows a picture of the experimental setup. In this scheme, the arrows indicate the direction of movement of the experimental stand’s mobile parts.

We measured the rise in the bone temperature during the drilling of the porcine femurs in the presence of the three types of surgical guides fabricated, as explained in the previous section. A schematic representation of the experimental setup used in this study is depicted in Figure 3.

During the drilling, the bone pieces were immobilized using a stainless-steel hardwood screw mounted on a specially designed stand (Figure 3). The stand enabled us to orient the bone surface of the target site horizontally, whereas the drilling proceeded vertically.

To exert a constant load on the drilling handpiece, we built a wooden stand with a rotating arm of 1.2 m in length (Figure 3). To ensure that a constant axial load of 2 kg-force (kgf) was applied [33], the position of a sliding weight (Figure 3, (5)) was adjusted before each experiment, and the force was measured using a hanging scale with a precision of 1.0 g. The handpiece, wrapped in a sheet of silicone rubber, was placed, in a well-defined position, into a channel carved in the rotating arm. It was fixed, using three wood screws, such that the drill bit was vertical when the rotating arm was horizontal.

Pieces of fresh porcine femoral bone were acquired on each day of measurement from a local supermarket. They were stored in physiological saline at 4 °C. Before the measurements, the bone pieces were placed, for at least one hour, in a water bath maintained at 25 °C.

Three measurements were performed for each piece of bone: one for each sort of surgical guide, taken in a random order to mitigate the impact of drill wear on the bone heating. After each measurement, the respective bone was placed back into the water bath for at least 10 min to dissipate the heat generated in the course of the drilling.

To test the thermodynamic efficacy of the guides, we recorded the temperature rise caused by drilling performed at 1500 rpm using a surgical osteotomy preparation drill—2.8 × 13.5 mm Simple Guide Plus, (Dentis, Daegu, Republic of Korea). The drilling parameters used in this work have been used in several studies of bone heating due to surgical drilling [33], including the one conducted by Misir et al. [15]. The use of standard drilling parameters was strongly recommended in the systematic review conducted by Möhlhenrich et al. [33].

The guide was mounted on a femur piece, such that its margin was aligned with that of the bone (Figure 3, picture inset), assuring that the drilling was performed vertically at a distance of 5 mm from the bone margin, while the bone’s surface was placed horizontally. All the surgical guides had 4 orifices, 1.5 mm in diameter, in the corners of their basal surface (the one that contacted the bone—as shown in Figure 2). We immobilized the guides on the bone using a stainless-steel wire of 0.2 mm in diameter. The drilling was performed under external cooling with saline solution at room temperature (25 °C), delivered at a rate of 40 mL/min. In the presence of the classical guide (Figure 2A) and the open-sleeve guide (Figure 2B), the irrigant was delivered from the nozzle of the physiodispenser, whereas, in the case of the guide with auxiliary cooling, the irrigation tubing was connected to the incorporated coolant channel (Figure 2D).

The bone temperature was measured, with an accuracy of 0.05 °C, using a Delta OHM HD 2108.2 digital thermometer (Delta OHM, Padua, Italy) equipped with a K-type thermocouple (Delta OHM, Italy). The thermocouple was inserted into the cortical bone, in a hole drilled perpendicularly to the direction of the osteotomy, in the middle of the cortical bone layer. The bottom of the temperature measurement slot was positioned 1 mm apart from the edge of the planned osteotomy drill path. For precise thermocouple placement, a 3D-printed cylindrical guide was used to limit the penetration of the drill into the cortical bone. Once the thermocouple was inserted into the hole, we filled the empty portion of the hole with pork fat to seal the thermocouple from the irrigating fluid.

### 2.3. Statistical Analyses

This paper presents the experimental results as mean ± standard deviation (SD). For the hypothesis testing, the level of statistical significance was set to *p* < 0.05. The statistical analyses and visualizations were performed using MedCalc version 20.015 (MedCalc Software Ltd., Ostend, Belgium).

We used violin plots to visualize the distribution of the recorded data, as well as to spot outliers.

The sample size, *n* = 14, was assessed using a statistical power analysis to ensure an 80% chance of detecting, at a significance level of 0.05, an effect size of 1 °C, assuming that the SD of the differences was 1.2 °C. The effect size and SD were determined from the preliminary data acquired on 5 bone samples for each guide. They were in good agreement with previous studies of bone heating during guided osteotomy [24,25].

To identify significant differences in the mean temperature increments during osteotomies with various surgical guides, we employed a one-way analysis of variance (ANOVA). Then, Scheffé’s post hoc test was used for pairwise comparisons.

Additionally, we conducted a Bland–Altman analysis to assess the temperature rise discrepancies between the osteotomies performed in the presence of different surgical guides. First, data points representing the differences were plotted against their means. Then, a solid horizontal line was added to represent the mean difference (also known as the bias) and dashed lines to indicate the 95% interval of agreement, delimited by the lower and upper limits of agreement (LLA and ULA, respectively; LLA = Mean − 1.96 SD, ULA = Mean + 1.96 SD). These values were specific to the sample, and their 95% confidence intervals (CIs) were represented with error bars, reflecting agreement for the entire population.

## 3. Results

**Figure 4:**
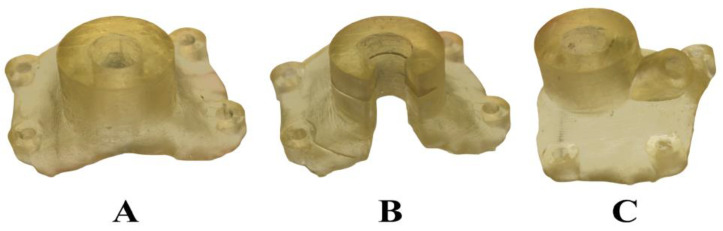
Photographs of the three types of surgical guides tested in this study. (**A**) The classical guide; (**B**) the open-sleeve guide; and (**C**) the guide with auxiliary cooling.

Photographs of the surgical guides fabricated using 3D printing from NextDent SG dental resin are shown in Figure 4. The classical guide (panel A) was designed to fit the lateral surface of the femoral piece shown in Figure 1. The open-sleeve guide (panel B) was obtained by eliminating part of the classical guide. The surgical guide with auxiliary cooling (panel C) was obtained by augmenting the classical guide with a connector for irrigation tubing. The different view angles, however, make it difficult to compare the sizes of the guides. Except for their different structural features (the lateral niche in Figure 4B and the coolant pipe in Figure 4C), the three guides were identical in size and design.

**Figure 5:**
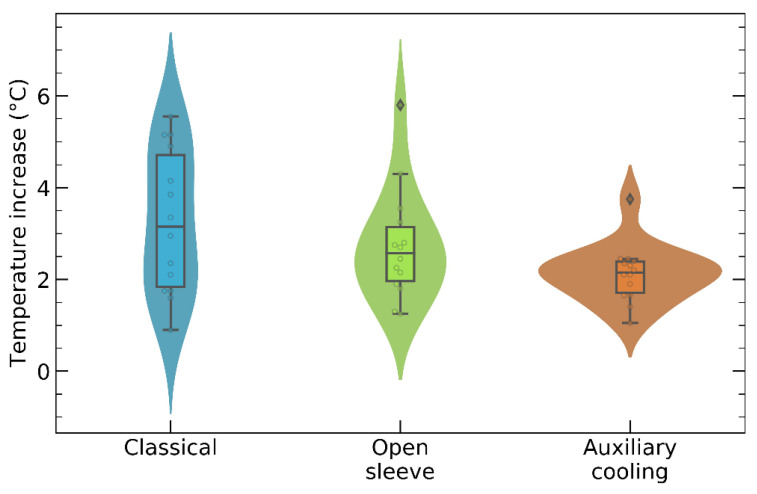
Violin plots of the maximal temperature increments recorded during osteotomies performed using the surgical guides from Figure 4.

Figure 5 presents violin plots of the data sets recorded during the 42 independent measurements conducted on 14 porcine femoral pieces. On each of them, one osteotomy was performed with each surgical guide, and the maximum temperature increments were recorded.

Individual data points are depicted as circular markers in Figure 5, whereas the probability density function is represented by the lateral profile of the corresponding violin plot. In each plot, the central box spans the interquartile range (IQR), defined as the difference between the third quartile (Q3) and the first quartile (Q1), whereas the horizontal segment that divides the box represents the median, or second quartile (Q2); 25% of the data points lie below Q1, 50% of them lie below the median, and 75% of them are below Q3. The vertical segments reach out to the points located at 1.5 × IQR or less from the box; points located beyond these segments are considered to be outliers (marked by diamond markers in Figure 5). Remarkably, none of the individual peak temperature rises exceeded the bone damage threshold of 10 °C.

**Figure 6:**
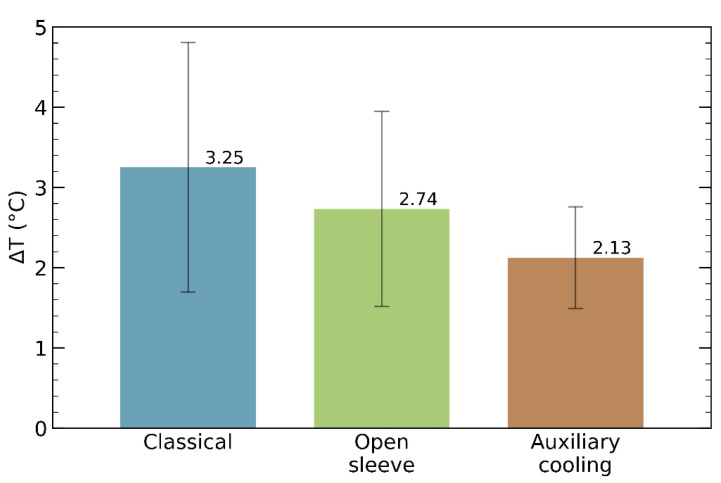
Bar plots of the mean values of temperature elevations incurred while the osteotomy was performed using different surgical guides. Error bars represent standard deviations.

Figure 5 and Figure 6 indicate that the drilling caused the highest rise in the bone temperature when the classical guide was mounted on the femoral piece. Although the open-sleeve guide enabled better cooling than the classical one, it was suboptimal. Indeed, the least bone heating was observed in the presence of the surgical guide with an incorporated coolant channel (Figure 4C). In Figure 6, the bars represent the mean values of the temperature increments, whereas the error bars show the corresponding SDs.

Nevertheless, the question arises of whether the differences between the mean temperature rises observed for the three surgical guides are statistically significant or not. To answer this question, we performed a one-way analysis of variance (ANOVA) test, obtaining a *p*-value of 0.056, which is marginally higher than 0.05. Hence, one cannot reject the null hypothesis that there are no statistically significant differences between the mean values.

**Figure 7:**
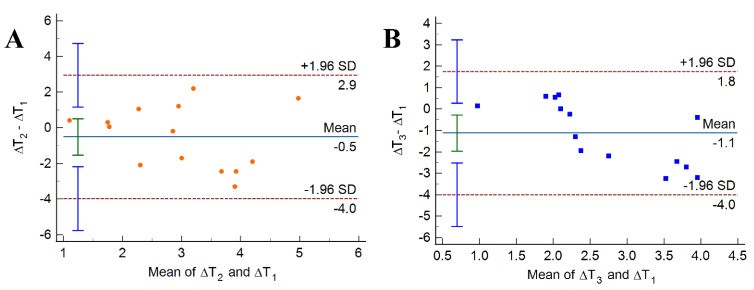
Bland–Altman plots of differences vs. means of temperature increments caused by osteotomies performed on porcine femurs in the presence of the classical guide (ΔT_1_), the open-sleeve guide (ΔT_2_), and the surgical guide with auxiliary cooling (ΔT_3_). (**A**) Open-sleeve compared to classical. (**B**) Auxiliary cooling compared to classical. In these plots, each marker corresponds to one bone specimen. The solid horizontal line, labeled “Mean”, represents the mean value of the differences, whereas the dotted horizontal lines delimit the 95% interval of agreement. Error bars represent 95% CIs.

The Bland–Altman plots from Figure 7 show the individual discrepancies between the temperature changes inflicted while the surgical drilling was conducted in the presence of the different guides. Differences between the corresponding temperature changes are plotted versus their mean values. Compared to the classical guide, the open-sleeve guide resulted in a negative bias of −0.5 °C (labeled as “Mean”) (Figure 7A), which suggests that the open-sleeve guide provided better cooling. Individual differences, however, were rather large; 95% of them are enclosed between the dotted horizontal lines that depict the limits of agreement.

The osteotomies performed while the guide with auxiliary cooling was in place resulted in a bias of −1.1 °C compared to the temperature rise observed while using the classical guide. Zero was not part of the 95% CI of the bias (represented by the green vertical error bar centered on the solid horizontal line from Figure 7B). Moreover, the interval of agreement is narrower in Figure 7B than in Figure 7A, which is consistent with the relatively small spread of the temperature increments observed in the case of auxiliary cooling.

## 4. Discussion

The problem of heat dissipation during implant site preparation has attracted increasing attention during the last two decades [33]. Understanding the factors that contribute to the temperature rise in the bone adjacent to the drill would enable clinicians to minimize cellular damage. Tens of studies have addressed this problem, albeit with a vast variety of study designs and materials, making comparisons difficult and meta-analyses inappropriate [33]. The use of surgical drill guides makes the problem of heat generation even more complex, since the guide masks the site of the osteotomy, impeding the access of the irrigation fluid. The solution evaluated in the present study consists of incorporating a coolant tube into the design of the surgical drilling guide, as proposed by Liu et al. [31].

Relying on a widely used dental implant simulation software, we designed three types of surgical templates to fit a representative piece of porcine femur. They were identical except for the guiding sleeve, which was cylindrical in the case of the classical guide, C-shaped in the case of the open-sleeve guide, and augmented with a lateral coolant access channel in the case of the guide with auxiliary cooling. Then, we built the guides from dental resin via 3D printing. Finally, we measured the temperature increments during osteotomies performed under a constant axial force. Regardless of the template design, all the temperature elevations remained below the critical threshold of 10 °C. Neglecting outliers, the peak temperature rise was 5.6 °C for the classical guide, 4.3 °C for the open-sleeve guide, and 2.5 °C for the guide with auxiliary cooling (Figure 5). Remarkably, not just the median of the temperature increments, but the IQR was also smallest for the guide with incorporated tubing, suggesting that it assured the best cooling and did so consistently. The same conclusion can be drawn by inspecting the bar graph in Figure 6: the mean value, as well as the SD, of the drilling-induced temperature rise was the smallest for the guide with auxiliary cooling. To our knowledge, the present study is the first to compare the thermodynamic performances of the open-sleeve design and the internal coolant duct design. It suggests that both of them favor coolant access, but the incorporated irrigation fluid channel ensures more effective cooling of the osteotomy site.

Nevertheless, the differences between the mean values of the temperature increments were marginally insignificant, presumably because of the large variance observed in the case of the classical guide, and/or the large biological variability of the employed in vitro model (porcine femur). Alternatively, the absence of a coolant exit channel might have contributed to the lack of a statistically significant difference [24]. Indeed, in the study conducted by Stocchero et al., the internal irrigation channel turned out to be ineffective [25], whereas a similar approach with an additional coolant exit channel resulted in significant reduction in the intraosseous temperature during guided drilling [24]. The above studies [24,25] shared the design feature that the surgical template closely fit each bone specimen. In contrast, in our work, the surgical guide was designed to fit one spot of a representative specimen and it was used in all the subsequent measurements. Since the guide did not wrap the femur tightly, it did not block the evacuation of the irrigation fluid that rinsed the burr’s entry point. Further research will be needed to elucidate whether the addition of a coolant exit channel makes cooling more effective, also in the case of surgical guides that do not touch the target site—as is usual in clinical applications [22,26].

Our evaluation of the cooling effectiveness was, in many respects, similar to that of Misir et al. [15]: we used the same drilling speed and axial load, also recommended in a systematic review of heat dissipation during dental implant site preparation [33], and we monitored the increase in bone temperature in the vicinity of the drilling site using a thermocouple inserted into a hole drilled perpendicularly to that of the osteotomy. Nevertheless, working with a less dense bone (porcine instead of bovine femoral pieces), we observed smaller temperature increments. The bovine femoral cortical bone model was also used by Teich et al. to validate the cooling performance of a surgical guide design with an internal coolant duct [26]. They too observed higher temperature increments of 21.8 °C in the presence of a classical surgical guide and only 6.52 °C for a guide with internally routed irrigation. Moreover, their paper also reported a clinical case study, reinforcing the message of previous clinical studies [22,23], in that the new design principles can readily be implemented in practice [26].

A recent comparative study [10] demonstrated statistically significant differences in the mean temperature rises in the presence of a cylindrical sleeve guide and a guide that did not limit the access of the irrigating fluid (0.82 °C and 0.3 °C, respectively). These were the averages of the temperature increments recorded in the cortical layer of bovine rib samples, at a depth of 1.5 mm and 1 mm from the edge of the osteotomy. The individual temperature elevations were smaller than 3.34 °C, within the safe zone, for both types of drilling templates. Classical and open-sleeve guides were compared in a vast study [12] performed on polyurethane foam blocks that mimicked two extreme bone densities, D1 and D4—according to the bone density scale devised by Misch [1]. The open-sleeve guides provided a smaller intraosseous temperature rise of 1.1 °C in D1 bone and 1.42 °C in D4 bone. Furthermore, the temperature of the irrigation fluid played a significant role, resulting in an average temperature drop of 0.96 °C when the coolant temperature was reduced from 21 °C to 5 °C. [12]. A comparative study of five different surgical template designs performed on bovine rib samples did not reveal statistically significant differences between the temperature elevations caused by guided osteotomies [16]. Nevertheless, it demonstrated that the open-sleeve guide provided more effective cooling than that of the closed-sleeve, occlusal splint design (on average, the corresponding intraosseous temperature elevations were 3.8 °C and 5.6 °C, respectively). Our study indicated that the open-sleeve design facilitates bone cooling, but less effectively than the one with an incorporated coolant channel.

There is a vast body of evidence suggesting that surgical templates do not shift the intraosseous temperature beyond the bone damage threshold and new designs can facilitate or guide the coolant toward the site of the osteotomy, resulting in a further reduction in the temperature rise caused by the surgical drilling. Nevertheless, theoretical investigations have suggested that this problem deserves further scrutiny. Due to practical reasons, the close proximity of the drill escapes experimental measurements. Thermocouples are typically placed at 0.5–1.0 mm from the anticipated periphery of the osteotomy, whereas infrared thermography has a limited resolution. Computer simulations indicate, however, that the most important bone heating occurs within 0.5 mm from the osteotomy’s margin [34]. Therefore, experimental investigations should be backed up by theoretical modeling to infer the spatial distribution of the intraosseous temperature [31,34].

This study has several limitations. The chosen experimental model (porcine femur), although relevant in what concerns cortical bone thickness, is less standardized than the bovine rib model [35]. Furthermore, the employed experimental model does not allow for discernment between the flapless and the open-flap approach. Additionally, the reported results are limited to a single-point measurement of the intraosseous temperature at the middle of the cortical bone layer. Further investigations will be needed to integrate theoretical modeling with temperature measurements at different depths in cortical, as well as trabecular bone.

A flapless guided osteotomy is the most appealing from a practical perspective because it involves little trauma, and thereby ensures fast healing. Unsurprisingly, it leads to the highest temperature elevation, because the drilling takes place under the surgical guide and a layer of soft tissue [13,19]. To assess the impact of the soft tissue, Jeong et al. conducted experiments on resin models of the mandible [13]. They incorporated a wood block in the edentulous portion of the model to mimic the alveolar bone and covered it with 2 mm thick silicone lining to represent the soft tissues. The maximum temperatures recorded at 6 mm beneath the top of the wood block and 0.5 mm from the periphery of the osteotomy were 32.6 °C in the flapless guided drilling and 31.3 °C in the open-flap guided drilling. Their difference was statistically insignificant [13].

Future studies could combine the bovine rib model [35,36] with computer simulations [34] to characterize the bone temperature field in the course of surgical drilling performed in the presence of different types of surgical templates. Besides multi-point temperature recordings based on thermocouples, the temperature field could also be probed using infrared thermography [33]. In addition, the bone heating caused by a single-step osteotomy could be compared with that encountered during sequential drilling, which is more common in a clinical setting. Even though bovine rib specimens possess most of the mechanical and thermal characteristics of alveolar bone, they lack circulation. Therefore, in vivo experiments conducted on animal models combined with theoretical investigations might provide further insights into the temperature rise at the edge of the osteotomy.

## Data Availability

All data acquired during this research are included in the article.
